# A Rare Case of Brodie’s Abscess in HIV Patient Caused by Mycobacterium kansasii

**DOI:** 10.7759/cureus.10153

**Published:** 2020-08-31

**Authors:** Kamna Bansal, Preethi Nambi

**Affiliations:** 1 Family and Community Medicine, Baylor College of Medicine, Houston, USA

**Keywords:** brodie's abscess, atypical mycobacteria, hiv, mycobacteria kansasii

## Abstract

We report a rare case of Brodie’s abscess caused by *Mycobacterium kansasii* (*M. kansasii*). Our patient is a 39-year-old male who presented with right foot pain a month after a new diagnosis of HIV infection. X-ray and MRI were done, and the diagnosis of Brodie's abscess was confirmed. Surgical debridement was done, and bone cultures grew *M. kansasii* after five weeks. Brodie’s abscess is a subacute form of osteomyelitis usually caused by *Staphylococcus*. Some other bacteria have been implicated in several case reports. To best of our knowledge, this is the first case of Brodie's abscess caused by *M. kansasii*. *M. kansasii* is the atypical mycobacteria causing infections in immunocompromised hosts as in HIV patients with low CD4 count. *M. kansasii* is usually associated with lung infections with rare extrapulmonary manifestations as in our case.

## Introduction

HIV patients with low CD4 count are prone to infections with atypical mycobacteria like *Mycobacterium kansasii *(*M. kansasii*). The incidence of *M. kansasii* infections in HIV patients may be as high as 532 per 100,000 population per year as compared to the general population where incidence is 0.5 per 100,000 population. *M. kansasii *often causes lung infections similar to *Mycobacterium tuberculosis*. Most HIV patients with infection with *M. kansasii *are associated with advanced immunosuppression with CD4 count <50 cells/μL. However, patients with comorbid lung pathology may get an infection at a higher CD4 count. Disseminated disease is uncommon in immunocompetent hosts, but it has been associated with HIV patients due to immunosuppressed state. Skeletal infections are rare with mycobacteria even in HIV patients. Musculoskeletal involvement usually happens after trauma or steroid injection causing monoarticular septic arthritis or tenosynovitis. Some cases of osteomyelitis associated with *M. kansasii *have been reported in immunocompromised patients. These usually involved the spine and skull. We present a case of a young HIV patient with Brodie's abscess of tibia caused by *M. kansasii*. To the best of our knowledge, it is the first case of Brodie's abscess caused by this *M. kansasii *reported in the literature.

## Case presentation

**Figure 1:**
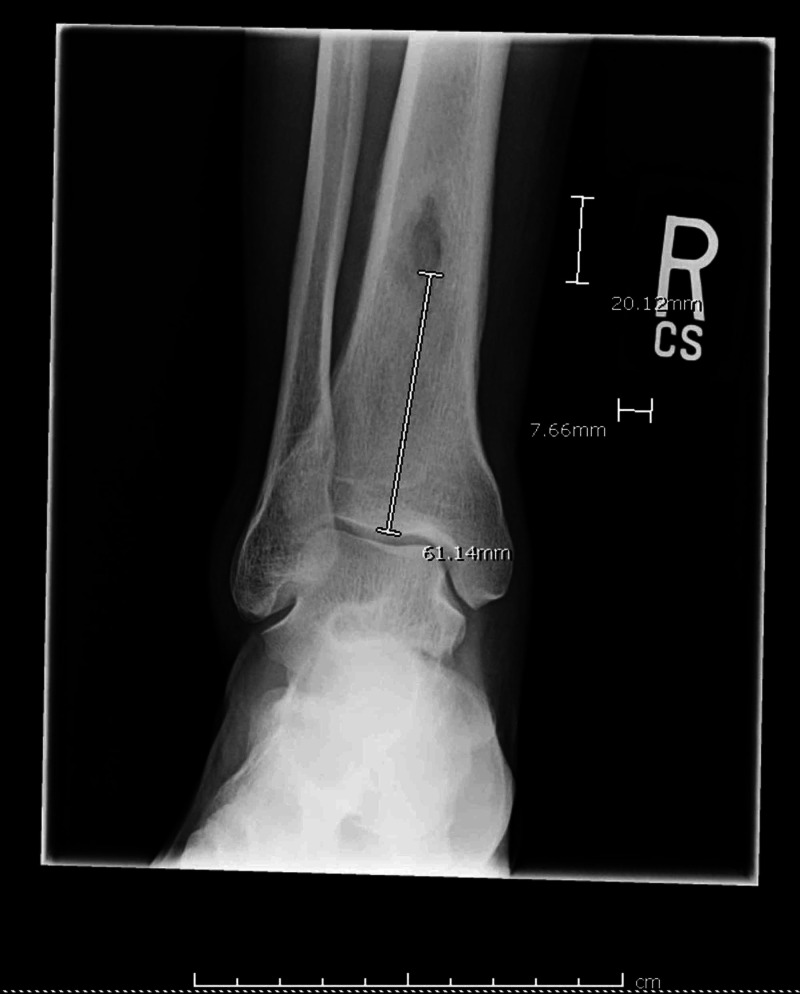
X-ray of the right ankle A 20-mm lesion seen in the distal tibia diaphysis was highly concerning for osteomyelitis with a Brodie's abscess given the aggressive periosteal reaction.

A 39-year-old male with HIV on antiretroviral therapy (ART) presented to a primary care physician with non-traumatic right ankle pain for about one-month duration. He was diagnosed with HIV about one month ago and ART (bictegravir 50 mg daily, emtricitabine 200 mg daily, and tenofovir alafenamide 25 mg daily) was initiated soon afterward. The last CD4 count about one month ago was 82 cells/μL (reference range 431-1,623 cells/μL). A right ankle X-ray (Figure 1) showed a 20-mm lesion in the distal tibia diaphysis concerning for osteomyelitis with a Brodie's abscess due to aggressive periosteal reaction.

**Figure 2:**
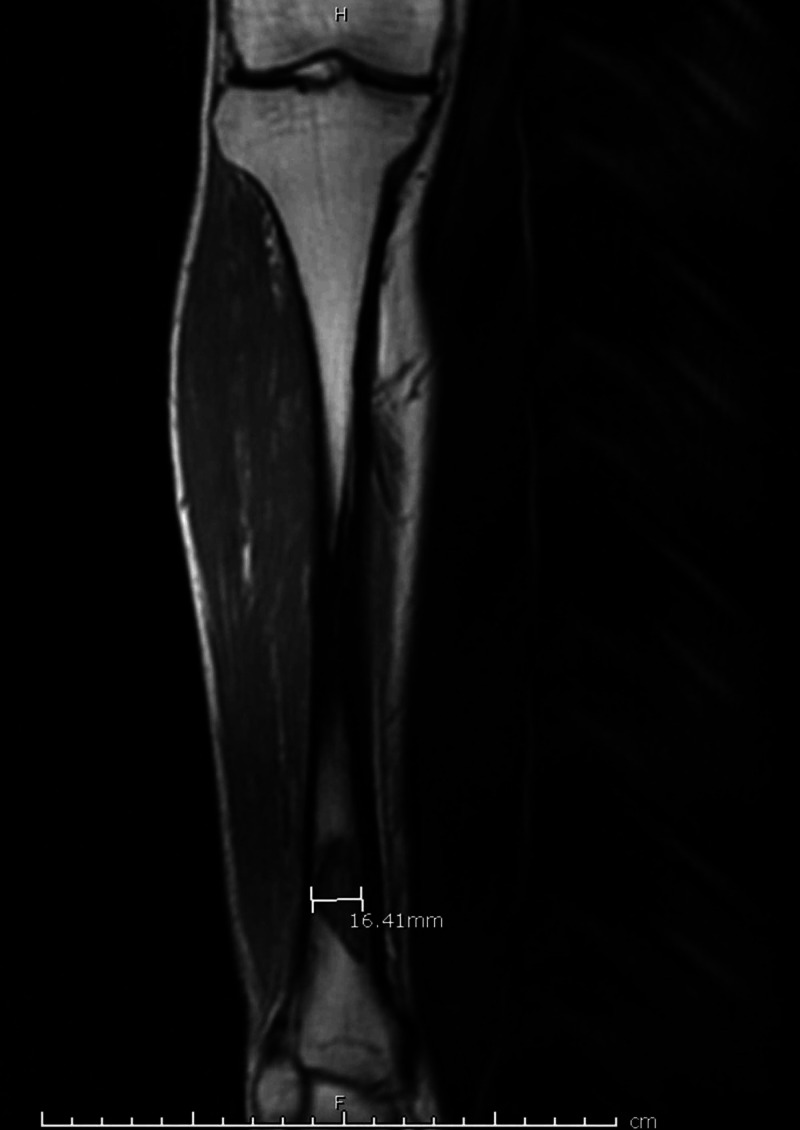
MRI of the right lower extremity (tibia and fibula) MRI shows osteomyelitis of the distal tibia with the formation of Brodie's abscess, which drains posteriorly into the soft tissues of the posterolateral tibia and fibula.

MRI of the right lower leg (Figure 2) was done and confirmed Brodie's abscess in posterior distal tibial diaphysis draining posteriorly into the soft tissues of the posterolateral tibia and fibula.

The patient was afebrile. Inflammatory markers were elevated: C reactive protein 2.5 mg/L (normal <1 mg/L), erythrocyte sedimentation rate was 26 mm/hr (normal 0 to <15 mm/hr), and white blood cell count 13.9 K/μL (normal 4.5-12 K/μL). The patient underwent surgery with debridement and irrigation. Initial cultures were negative. He was started on broad-spectrum antibiotics (sulfamethoxazole-trimethoprim DS 800-160 mg two tablets two times a day and amoxicillin-clavulanate 875-125 mg one tablet two times a day) and discharged home on postoperative day 6. Pathology showed granulomatous osteomyelitis highly suspicious for tuberculosis (TB). Acid-fast bacilli (AFB) and Grocott's methenamine silver (GMS) stains were negative. QuantiFeron®-TB Gold Plus test was positive (reference - negative). He had no evidence of lung TB on a chest X-ray. Given high suspicion for TB osteomyelitis, he was started on four-drug antitubercular therapy (rifabutin 300 mg daily, isoniazid 300 mg daily, ethambutol 1,200 mg daily, pyrazinamide 1,500 mg daily, and pyridoxine 50 mg daily), and broad-spectrum antibiotics were discontinued. His ART regimen was modified to avoid drug interactions. His ART regimen was changed to abacavir 600 mg daily, dolutegravir 50 mg daily, and lamivudine 200 mg daily. About five weeks later, his bone cultures came back positive for *M. kansasii*. His regimen was adjusted according to sensitivities (rifabutin 300 mg daily, isoniazid 300 mg daily, ethambutol 1,200 mg daily, and pyridoxine 50 mg daily were continued, and pyrazinamide was discontinued). He was advised to continue with medications for nine months. 

## Discussion

Brodie’s abscess was first reported by Sir Benjamin Brodie in 1832 [1]. It is subacute osteomyelitis associated with the accumulation of pus. Onset is usually insidious, and systemic signs and symptoms of infection are usually lacking [2]. Hematogenous spread often causes seeding of bacteria in the bone. Trauma has been implicated as a cause in some cases. Brodie’s abscess is usually located in the metaphysis of the tubular bones of the lower extremity. Tibia is the most common bone to be infected [3]. Less frequently, it can happen in other tubular bones or in the diaphyseal region as in our case. Brodie’s abscess is a contained infection surrounded by sclerotic bone, and this is the cause for little systemic response. 

The patient usually presents with pain. A physical exam may reveal swelling and tenderness. As the onset is slow and it does not have obvious symptoms, diagnosis is often delayed by weeks and even months. Imaging is considered the gold standard for diagnosis of Brodie’s abscess. X-rays are often done first and may reveal solitary lesion in the intramedullary region with well-defined sclerotic margins [4]. MRI often follows plain films and is highly sensitive for diagnosing Brodie’s abscess. It may show ‘penumbra sign’ which is a typical four-layered target appearance: a center, an inner ring, an outer ring of fibrotic reaction, and a peripheral halo of bone marrow edema [5]. 

It is frequently treated by surgical debridement followed by antibiotics. Culture and tissue debridement play a crucial role in identifying the causative organism. *Staphylococcus aureus* (30%-60%) accounts for most of the infections causing Brodie’s abscess. Other common pathogens are coagulase-negative *Staphylococcus*, *Pseudomonas*, and *Klebsiella*. About 25% of the cultures are negative and no organism can be identified [6]. *M. kansasii *is the most pathogenic atypical mycobacteria, as most of the culture-positive cases present with clinical manifestations. Tap water is the most common source and mode of infection is aerosolization in most cases. Immunocompromised status as in HIV with low CD4 count makes a patient susceptible to infections with *M. kansasii*. *M. kansasii *most commonly involves lungs, and extrapulmonary manifestations are rare. The extrapulmonary disease usually involves the skin, lymph nodes, and genitourinary and musculoskeletal systems. Musculoskeletal manifestations usually present with tenosynovitis and septic arthritis [7]. Brodie’s abscess with *M. kansasii *as in our case has not been reported in the literature. Treatment of extrapulmonary manifestations often requires a longer course of antibiotics up to 12 months. Concurrent ART therapy improves prognosis in HIV patients. 

## Conclusions

*M. kansasii* is the second most common atypical mycobacteria that cause pulmonary and disseminated infections after *Mycobacterium *avium complex. *M. kansasii* is an unusual pathogen presenting as osteomyelitis and Brodie’s abscess. Atypical mycobacteria causing bone infections usually follow trauma but hematogenous transmission can happen as in our case. Both tissue pathology and bone culture results are imperative in making a correct diagnosis. The presence of granulomas in tissue pathology and the positive QuantiFERON-TB Gold test helped in narrowing down mycobacteria as the likely causative organism. Positive bone cultures helped narrow down the causative organism to be *M. kansasii.* It is important to consider atypical mycobacterial infections as a differential when evaluating patents with bone infections and abscesses, especially in immunocompromised individuals.

## References

[1] Brodie BC (1832). An account of some cases of chronic abscess of the tibia. Med Chir Trans.

[2] Harris NH, Kirkaldy-Willis WH (1965). Primary subacute pyogenic osteomyelitis. J Bone Joint Surg Br.

[3] Stephens MM, MacAuley P (1988). Brodie's abscess. A long-term review. Clin Orthop Relat Res.

[4] Lopes TD, Reinus WR, Wilson AJ (1997). Quantitative analysis of the plain radiographic appearance of Brodie's abscess. Invest Radiol.

[5] Moser T, Ehlinger M, Chelli Bouaziz M, Fethi Ladeb M, Durckel J, Dosch JC (2012). Pitfalls in osteoarticular imaging: how to distinguish bone infection from tumour?. Diagn Interv Imaging.

[6] van der Naald N, Smeeing DPJ, Houwert RM, Hietbrink F, Govaert GAM, van der Velde D (2019). Brodie's abscess: a systematic review of reported cases. J Bone Jt Infect.

[7] Johnston JC, Chiang L, Elwood K (2017). Mycobacterium kansasii. Microbiol Spectr.

